# *In-Situ* observation of local atomic structure of Al-Cu-Fe quasicrystal formation

**DOI:** 10.1038/s41598-018-37644-x

**Published:** 2019-02-04

**Authors:** Hadi Parsamehr, Ying-Jiu Lu, Tzu-Ying Lin, An-Pang Tsai, Chih-Huang Lai

**Affiliations:** 1 Department of Material Science and Engineering, National Tsing Hua University, Hsinchu, 30013 Taiwan; 2 National Synchrotron Radiation Research Center, Hsinchu, 30076 Taiwan; 3 Department of Electrical Engineering, Tokyo University of Science, Tokyo, Japan; 4 Institute of Multidisciplinary Research for Advanced Materials, Tohoku University, Sendai, 980-8577 Japan; 5 National Institute for Materials Science, 305-0047 Tsukuba, Japan

## Abstract

The phase and local environment, neighbouring atoms and coordination numbers (CN), for an Al-Cu-Fe multilayer were studied during heating (to 800 °C) and cooling (to room temperature) processes using *in-situ* X-Ray diffraction (XRD) and *in-situ* X-ray absorption spectroscopy (XAS) techniques to investigate the formation of Al-Cu-Fe quasicrystals (QCs). *In-situ* XRD clarified the transition of the ω-Al_7_Cu_2_Fe phase to a liquid state at the high temperature which transformed into the QC phase during cooling. The *in-situ* XAS showed a relatively small shift in distance between Cu-Al and Fe-Al during the phase evolution from RT to 700 °C. The distance between Cu-Cu, however, showed a significant increase from ω-phase at 700 °C to the liquid state at 800 °C, and this distance was maintained after QC formation. Furthermore, the CN of Fe-Al was changed to N = 9 during cooling. Through our observations of changes in CN, atomic distances and the atomic environment, we propose the local structural ordering of the quasicrystalline phase originated from a liquid state via ω-phase. In this study, we give a clear picture of the atomic environment from the crystalline to the quasicrystalline phase during the phase transitions, which provides a better understanding of the synthesis of functional QC nanomaterials.

## Introduction

Quasicrystals (QCs) lack translational symmetry but retain long-range order in the spatial arrangement of atoms, the pattern of which never repeats^1^. These unique materials were initially believed to only be formed in a metastable state as their internal energy lays in an intermediate state between a crystal and an amorphous solid^2^. Multiple examples of stable QCs have been found^3^, notably Al-Cu-Fe QCs^4^. The Al-Fe-Cu system has been extensively studied due to the availability and affordability of the system’s components^5^. However, the mechanism of local atomic ordering in the transition from a crystalline to a quasicrystalline state is not well understood. A more thorough understanding of the formation, phase stability, structure, and the properties of this phase is necessary to exploit QC materials for industrial applications, such as coatings that can significantly increase surface corrosion durability, mechanical strength and wear resistance. Thus, structure characterisation throughout the formation of the QC is crucial to achieve these aims. Previous research has utilised techniques such as X-ray diffraction (XRD) and neutron scattering to study QC phases and structures^4,6–9^; however, due to their weak sensitivity, such techniques cannot capture the peculiarities of the local structure. Transmission electron microscopy (TEM) has also been used^10–16^, but this technique can only examine a small part of a specimen. On the contrary, the X-ray absorption spectroscopy (XAS) techniques can probe a much more substantial area. The main advantage of XAS over other techniques is that it provides a more detailed look at the number of neighbouring atoms, the type of atoms in the immediate environment of the absorbing atom, and the distances between central and neighbouring atoms. However, previous studies on QCs using XAS are quite limited and have been carried out in an *ex-situ* manner in which the changes to phase composition and structure under the heating and cooling conditions cannot be observed^17–22^. The coordination numbers (CN) and atomic distances for QCs and other crystal structures for Al-Cu-Fe have been observed at room temperature^23^; however, due to the *ex-situ* nature of this study, the CNs and atomic distances of different phases during the phase evolution and during the liquid state preceding QC formation have yet to be determined. To understand the properties during these phase evolutions up to QC formation, we employed *in-situ* XAS techniques, which can contribute valuable information regarding structural characterisation throughout formation.

In this paper, we apply the X-ray absorption fine structure (EXAFS) and X-ray absorption near edge structure (XANES) techniques in an *in-situ* study of QC thin film formation with the aim of determining the local mechanism responsible for the transition from a multilayer structure to a quasicrystalline state. Here, a multilayer Al-Cu-Fe structure (Si/SiO_2_/Al_2_O_3_/Al/Cu/Fe/SiO_2_) was annealed with a maximum temperature of 800 °C before being cooled back down to room temperature (RT). Thin films were used due to their notable advantages over bulk and powder samples, namely, their multilayer structure enables a clear picture of how the individual elements interact at different temperatures and how phases change during these temperature shifts^24^. Since annealing time and treatment methods have been shown to be crucial to the final phase evolution of the Al-Cu-Fe QC system^2–4,25^, EXAFS data were collected at different temperatures throughout the heating and cooling processes to capture the phase changes between Al, Cu and Fe. By combining the XANES and EXAFS data, we can determine interatomic distance, neighbouring atoms and CN for Cu and Fe as the QC forms.

## Materials and Methods

### Thin film preparation and characterization

**Figure 1:**
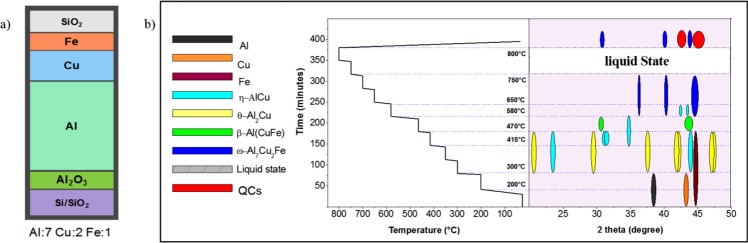
(**a**) Multilayer structure (Si/SiO_2_/Al_2_O_3_/Al/Cu/Fe/SiO_2_; (**b**) Heating-cooling profile for *in-situ* measurements vs phase evolution for Al-Cu-Fe from *in-situ* XRD.

Thin film depositions were made in a sputtering system equipped with five targets including elemental targets of Al, Cu and Fe. The Al–Cu–Fe films were deposited using Al (99.99% purity), Cu (99.99% purity) and Fe (99.99% purity) with circular targets ø3 inches in size. The thickness of the Al and Cu targets was 5 mm; a thinner target of 1.6 mm thickness was used for Fe. The background pressure was approximately 6 × 10^−8^ Torr, and no heat was applied during the depositions. Ar (99.999% purity) was used as the sputtering gas in all depositions with a sputtering pressure of 3 mTorr for depositions on cleaned Si/SiO_2_ substrates. The substrates were rotated at a rate of 50 rpm to improve the deposition uniformity. The deposition rates were 0.133 nm/s, 0.131 nm/s and 0.083 nm/s for Al, Cu and Fe, respectively. The layers were deposited with a total thickness of 1500 nm. The sequence of the samples used for both the *in-situ* XRD and *in-situ* EXAFS measurements was as follows: Si/SiO_2_/Al_2_O_3_/Al (1050 nm)/Cu (300 nm)/Fe (150 nm)/SiO_2_ (Fig. 1a). The *in-situ* XRD (HTXRD 6000 Shimadzu) measurements were used to collect the phases transition from RT to 800 °C and after cooling at RT. An XRD-1500 High-Temperature Oven (ADC Inc.) was used as an *in-situ* XAS chamber. The sample was heated in a vacuum condition, which was produced by a turbo pump with the pressure decreased to 5 × 10^−7^ Torr to avoid sublimation and oxidation. The heating-cooling profile was same for the *in-situ* XAS and *in-situ* XRD (Fig. 1b) and the average heating rate was 20 °C/min while the cooling rate was 50 °C/min. We also carefully checked if the sample was oxidized after the heat treatment. XANES spectra for Cu and Fe (Supplementary Fig. 1) as well as XPS data (Supplementary Fig. 2) for all three elements all reveal that no significant oxidation was observed after annealing.

The *in-situ* XRD measurements showed different phase transitions during the heating and cooling. A summary of the *in-situ* XRD data and the appearance of the different phases at different temperatures are shown in Fig. 1b. These phase transitions will be discussed in the following section.

### X-ray absorption measurements and analyses

X-ray absorption measurements were carried out with synchrotron radiation using the EXAFS facilities installed at the National Synchrotron Radiation Research Center, Hsinchu, Taiwan. The XAS measurements above the K-edges of Cu (8979 eV) and Fe (7112 eV) were performed with the BL17C1 beamline. In this study, the EXAFS spectra were analysed using the IFEFFIT^26^ software package for calculating amplitudes and phases. IFEFFIT, an interactive program for EXAFS analysis, corrects for instrumental effects, normalises unit edge steps, interpolates k-space, subtracts background interference and performs Fourier transformation^27^.

To have a better fitting, there are five variable internal parameters in EXAFS equation including N (coordination number), amplitude reduction factored (0.9 ≤ S_0_^2^ ≤ 1), the mean square variation in path length known as disorder term (σ^2^), the change in energy from theoretical data (ΔE ≤ 3 eV) and the change in interatomic distance(ΔR). The first three parameters (N, S_0_^2^ and σ^2^) known as the amplitude of the EXAFS oscillations and the ΔE and ΔR are the phase of oscillations^28^. The error bars for the atomic distance is related to the fitting parameter.

## Results and Discussion

A multilayered Al-Cu-Fe thin film was studied by *in-situ* XRD to observe the phase evolutions at specific temperatures during the heat treatment and cooling process. In addition, *in-situ* XAS was used to understand the local environment of Cu and Fe when these different phases were present. A single multilayer sample structure was prepared, and this sample was divided into several identical pieces for the *in-situ* XAS and the *in-situ* XRD experiments. The paper reports the results of a single *in-situ* XAS and a single *in-situ* XRD experiment with all data collected continuously at different temperatures. The data are quite reproducible as long as the thicknesses, heat treatment and layer sequences are same as detailed in the present work. For the *in-situ* XAS experiment, five scans were made at one temperature of the same sample within a single experiment, and the data from these scans were averaged to ensure accurate results at each of the temperature points.

The XANES-EXAFS spectrum related to the Cu K-edge (8979 eV) and Fe K-edge (7112 eV) were taken to observe the numbers of neighbouring atoms for Cu and Fe, the types of atoms in their local environments, and the distances between central and neighbouring atoms. The particulars of the EXAFS procedure used here have been described in detail elsewhere^29,30^. Throughout the *in-situ* XRD analysis, the phase transitions can be observed, as shown in Fig. 1b. Here, to clarify the interactions between Al, Cu and Fe, we divided these phase transitions into the following stages: RT to 350 °C, 350 °C to 470 °C, 470 °C to 700 °C, 700 °C to 800 °C, and the cooling from 800 °C to RT.

### FDMNES simulation

The XAS spectra of all the phases, identified by *in-situ* XRD, were simulated by Finite Difference Method (FDM) in FDMNES to verify the XAS experimental data. The FDM, introduced by Yves Joly to calculate the potential in the interstitial region^31^, is based on the Schröodinger equation to calculate X-ray absorption near-edge structure (XANES) spectra in the EXAFS. This method can be used to find the best fit for XANES. With FDMNES, the spectra of real and virtual absorption can be calculated. The crystal structure and symmetry for each phase for Cu and Fe K-edges with the range of 3 Å to 8 Å in the cluster radius were calculated by FDMNES to find the optimum cluster radius size from the simulation which can be used to compare to the experimental data from XAS. The optimum cluster size can be achieved when the trend of the graph with increasing the size of the cluster remains unchanged. The radius of the cluster needs to be sufficiently large in order to accurately calculate the final photo-excited state^32,33^. The optimum cluster sizes for the Cu K-edge from the FDMNES simulation for Cu, η-AlCu, θ-Al_2_Cu, β-Al (Cu, Fe) and ω-Al_7_Cu_2_Fe phases are 6 Å, 5 Å, 5 Å, 6 Å and 3 Å, respectively. As for the Fe K-edge, the optimum cluster sizes for Fe, β-Al (Cu, Fe) and ω- Al_7_Cu_2_Fe phases are 6 Å, 5 Å, and 3 Å, respectively (see Supplementary Fig. 1).

### Formation of Al-Cu binary phase

**Figure 2:**
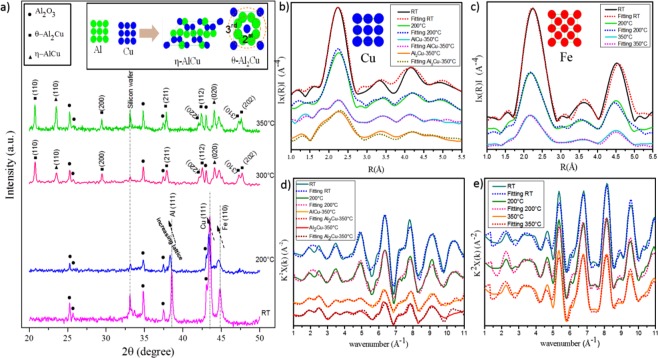
*In-situ* XRD graph form RT to 350 °C and the schematic of η-AlCu and θ-Al_2_Cu that formed from interaction of Al and Cu (**a**); the modulus of the Fourier transform |F(R)| of the EXAFS function of FCC Cu, BCC Fe, η-AlCu and θ-Al_2_Cu for the local environment of Cu (**b**) and Fe (**c**); experimental EXAFS function χ(k)k2, measured from RT to 350 °C above the Cu (**d**) and Fe (**e**) K-absorption edges.

The phase evolution of the Al-Cu-Fe multilayer in the initial stage of the heating process, from RT to 350 °C, was captured by *in-situ* XRD (Fig. 2a). At this stage, both η-AlCu and θ-Al_2_Cu are present. From the graph, we can observe that the Al, Cu and Fe lattices started to expand at 200 °C. This expansion enabled an interaction between Al and Cu as well as the formation of η-AlCu and θ-Al_2_Cu with different orientations at 300 °C. θ-Al_2_Cu appeared in (110), (200), (211), (310) and (202) orientations, and the intensity of these orientations increased when the temperature reached 350 °C. Besides θ-Al_2_Cu, η-AlCu was also seen in (110) and (020) orientations, and these orientations grew as the temperature increased. The insert in Fig. 2a provides a schematic diagram of η-AlCu (space group: 12, PEARSON mC20; lattice parameters: a = 12.15076 Å, b = 4.06235 Å, c = 6.90084 Å and β = 55.083°) and θ-Al_2_Cu (space group: 140, PEARSON tI12; lattice parameters: a = b = 5.57416 Å, c = 4.77381 Å).

### The XAS analysis for Al-Cu binary phase

The Fourier Transforms (FT) of the EXAFS signals for Cu and Fe are illustrated in Fig. 2b,c, respectively. The EXAFS oscillations, k2χ (k), obtained after standard data reduction, are shown in Fig. 2d,e as a function of the photoelectron wave vector k (the normalised experimental EXAFS spectra). The FTs of k2χ (k) were obtained in the wavelength range from 1 to 11 Å−1 for the Cu and Fe K-edges. The FT peak position is related to the distance between the target atom and its respective neighbours, while the peak area corresponds to the occupation of the neighbouring shells. A distinguished peak around 2 Å can be seen in both graphs from the first neighbour shell. Other peak positions are related to the neighbours in shells beyond the first shell. An FEFF model was then applied to the EXAFS data. To compare the CN, and the atomic distances, R, in the first coordination shell around Cu and Fe atoms in the different phases, the model within the 1.5–4.7 Å range were back-transformed to k-space. By using the best fit to match the XAS experimental data, the average distance of neighbouring atoms and the CNs for the principal shells of the atomic neighbours surrounding Cu and Fe could be collected for the different phases at different temperatures. The XAS data for the sample during the heating process for the FCC Cu, BCC Fe, η-AlCu and θ-Al_2_Cu from RT to 350 °C is shown in Fig. 2b–e. Notably, our results correspond with reference data at specific temperatures (Supplementary Table 1).

### Formation of Al-Cu-Fe ternary phase

**Figure 3:**
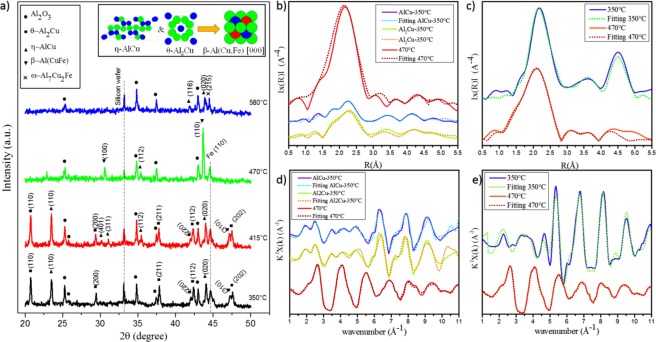
*In-situ* XRD graph form 350 °C to 580 °C and the schematic of the β-Al(Cu, Fe) that formed from the θ-Al_2_Cu and the η-AlCu with Fe interaction (**a**); the modulus of the Fourier transform |F(R)| of the EXAFS function of η-AlCu, θ-Al_2_Cu and β-Al(Cu, Fe) for the local environment of Cu (**b**) and Fe (**c**); experimental EXAFS function χ(k)k2, measured from 350 °C to 470 °C above the Cu (**d**) and Fe (**e**) K-absorption edges.

As the temperature increased from 350 °C to 470 °C, additional phases were observed. A β-Al (Cu, Fe) phase formed between Al, Cu and Fe in (100) and (110) orientations when the temperature reached 470 °C. During this stage, an XRD scattering was collected at 415 °C. At this temperature, the phases were almost identical to those seen at 350 °C except for two new orientations of η-AlCu phase ((401) and (311) orientations). However, θ-Al_2_Cu phase completely disappeared when the temperature reached 470 °C, and just a (211) orientation of η-AlCu phase remained. β-Al (Cu, Fe) phase remained until the temperature reached 580 °C, at which point a ω- Al_7_Cu_2_Fe phase appeared. Besides ω- Al_7_Cu_2_Fe phase, η-AlCu phase was also seen in (116) and (020) orientations, and the Fe peak completely disappeared (Fig. 3a). β-Al (Cu, Fe) phase is reported to have a CsCl structure at room temperature with a = 29.09Å^34^ and PEARSON cP2 (221). The schematic diagram can be seen in Fig. 3a.

### The XAS analysis for the Al-Cu-Fe ternary phase (470 °C to 700 °C)

To compute the distance between the environment atoms and target atoms, specifically the local environment and CN for β-Al (Cu, Fe) phase, XAS data was collected at 470 °C. Figure 3 shows the radial distance for Cu and Fe with neighbouring atoms as well as the normalised experimental EXAFS spectra at 470 °C for Cu and Fe. From Fig. 3b, we can see a sharp intensity of β-Al(Cu, Fe) phase at 470 °C compared to 350 °C for the Cu K-edge. Extracting the parameters from the fitting of the experimental data at 470 °C requires the reference of the specific crystal structure, which is undocumented for the β-phase at 470 °C. Thus, we used the database of the cubic CsCl but changed the lattice parameters to the β- phase (29.09 Å) to start our simulations. In addition, we calculated the cell volume and density of the β-phase for the specific composition (Al50Cu40Fe10)^35^, and these parameters were used to establish the best fit for the model (Fig. 3b–e). After making these modifications, the XAS data were analysed by the aforementioned method. The CN and the distance of atomic neighbours surrounding Cu and Fe centres are shown in Table 1.

**Table 1:** Average bond distances for the β-Al (Cu, Fe) and CN s for the principal shells of atomic neighbours surrounding Cu and Fe centers.

K-edge	type of atom	N	R*(Å)	K-edge	type of atom	N	R*(Å)
β-phase 470 °C (Cu)	Cu-Al	8	2.582 ± 0.0104	β-phase 470 °C (Fe)	Fe-Al	8	2.582 ± 0.0104
	Cu-Fe	6	2.950 ± 0.0106		Fe-Cu	6	2.950 ± 0.0107
	Cu-Fe	12	4.059 ± 0.0123		Fe-Cu	12	4.059 ± 0.0123

(R*: atomic distance to neighbouring atom from the experimental data, N: CN).

**Figure 4:**
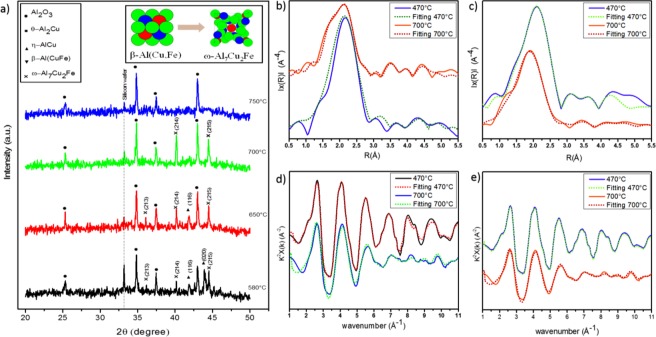
*In-situ* XRD graph form 580 °C to 750 °C and the schematic diagram of ω- Al_7_Cu_2_Fe that formed from the β-Al(Cu,Fe) (**a**); the modulus of the Fourier transform |F(R)| of the EXAFS function of β-Al(Cu,Fe) and ω- Al_7_Cu_2_Fe e for the local environment of Cu (**b**) and Fe (**c**), respectively; experimental EXAFS function χ(k)k2, measured from 470 °C to 700 °C above the Cu (**d**) and Fe (**e**) K-absorption edges.

The observation of phase changes continued as the sample entered the final stage of the heat treatment to the maximum temperature of 800 °C. When the temperature was increased to 650 °C, the intensity of ω- Al_7_Cu_2_Fe with (215) and (214) orientations grew, and η-AlCu with (020) orientation disappeared. Although η-AlCu with (116) orientation was still present at 650 °C, it also completely disappeared at 700 °C. At 700 °C, the only phase observed was ω- Al_7_Cu_2_Fe with (215) and (214) orientations. XAS data were collected at 700 °C (Fig. 4) to characterise the atomic environment of the ω-phases that were present in the XRD data at this point. The same methods described above were used to analyse the R-space (Fig. 4b,c) and k-space (Fig. 4.d,e). From these graphs, we can see the changes in R-space and k-space for Cu and Fe at 700 °C. The distance, the neighbouring environment and CN for the ω-phases can be seen in Table 2. Our fitting correlates well with the experimental data at 700 °C.

**Table 2:** Average bond distances and CNs for the principal shells of Cu and Fe in the ω-phase at 700 °C, liquid phase at 800 °C and QC phase at RT.

K-edge	Type of atom	N	R*(Å)	K-edge	Type of atom	N	R*(Å)	K-edge	Type of atom	N	R*(Å)
ω- phase at 700 °C (Cu)	Cu-Al (1)	2	2.533 ± 0.003	Liquid phase 800 °C (Cu)	Cu-Al (1)	2	2.441 ± 0.009	QC- phase at RT (Cu)	Cu-Al (1)	2	2.412 ± 0.019
	Cu-Cu (2)	2	2.545 ± 0.007		Cu-Al (2)	2	2.575 ± 0.011		Cu-Al (2)	2	2.489 ± 0.027
	Cu-Al (3)	4	2.579 ± 0.019		Cu-Al (3)	2	2.590 ± 0.023		Cu-Al (Fe) (3)	2	2.590 ± 0.010
	Cu-Al (4)	2	2.668 ± 0.022		Cu-Cu (4)	2	2.648 ± 0.017		Cu-Cu (4)	2	2.619 ± 0.021
		—	—		Cu-Al (5)	2	2.875 ± 0.030		Cu-Al (5)	2	2.721 ± 0.036
ω- phase at 700 °C (Fe)	Fe-Al (1)	5	2.441 ± 0.016	Liquid phase 800 °C (Fe)	Fe-Al (1)	5	2.589 ± 0.015	QC at RT (Fe)	Fe-Al (1)	9	2.489 ± 0.016
	Fe-Al (2)	4	2.473 ± 0.027		Fe-Al (2)	4	2.713 ± 0.023				

(R*: atomic distance to neighbouring atom from the experimental data, N: CN).

### Liquid state and formation of quasicrystal during cooling down

**Figure 5:**
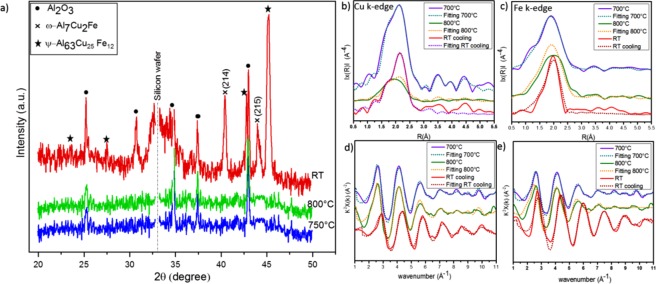
*In-situ* XRD graph from 700 °C to RT (**a**); the modulus of the Fourier transform |F(R)| of the EXAFS function of ω-Al7Fe2Cu, liquid phase and QC for the local environment of Cu (**b**) and Fe (**c**); experimental EXAFS function χ(k)k2, measured from 700 °C to RT above the Cu (**d**) and Fe (**f**) K-absorption edges.

As the temperature reached 750 °C, no metallic phases appeared in the XRD graph, with the exception of the substrate material (Al_2_O_3_) (Fig. 5a). This could be indicative of the sample having reached a liquid state, as has been observed in a previous study^36^. EXAFS has been reported to be an ideal technique to examine the sample in the liquid phase for investigating the environment of specific elements in a liquid^37,38^. EXAFS has been utilised to investigate the structure of amorphous systems and liquid state by measuring and comparing the EXAFS spectra in terms of the existence of considerable short-range order^39–42^. To fit the EXAFS spectra of the liquid state, the crystalline counterparts have been used to fit the atomic distance and atomic environment around the center atoms of the liquid state.

### XAS analysis for the Liquid state and quasicrystal

The XAS data collected during the final stage of the heat treatment and the cooling process are presented in Fig. 5. A decrease in the amplitude of the maxima of |F(R)| was observed for the sample at 800 °C compared to ω-Al7Fe2Cu at 700 °C in the range 1.0–2.8 Å around Cu atoms (Fig. 5b), which is suggestive of structural disordering of the nearest environment of Cu. Despite this difference in amplitude, the k-space graphs are similar (Fig. 5d). On the other hand, the amplitude around Fe did not change significantly in |F(R)|; the graphs in k-space were similar (Fig. 5b,e). To extract the XAS parameters in the liquid state obtained at 800 °C, we used the ω-phase crystal structure as a fitting model to discern the R* and N in the liquid state. The distance, neighbouring and CNs for Cu and Fe for the ω-Al7Fe2Cu at 700 °C (ω-phase) and 800 °C (liquid state) are shown in Table 2.

A comparison of these data shows that, in the first shell, Cu has the nearest distance with Al (2.533 Å) in ω- Al_7_Cu_2_Fe at 700 °C. At 800 °C, the distance of Al-Cu decreased to 2.441 Å, which indicates a stronger bond between Cu and Al in the first shell. In the second shell, the Cu-Cu atomic distance with N = 2 increased from 2.545 Å (at 700 °C) to 2.648 Å (at 800 °C). Moreover, the Cu-Cu bonding was in the second shell for the ω- Al_7_Cu_2_Fe at 700 °C, but when the sample reached the liquid state, a significant change in atomic distance was observed, and the Cu-Cu bonding was relocated to the forth shell. This observation suggests a weaker interaction between Cu-Cu in the liquid state compared to the crystal phase. Besides the relocation of the Cu-Cu shell, the Cu-Al in the third shell for ω- Al_7_Cu_2_Fe at 700 °C had an atomic distance of 2.579 Å with the CN 4 (N = 4), which split into two shells (second and third) at 800 °C with the equal CNs (N = 2) and distances of 2.575 Å and 2.590 Å, respectively.

A similar change in the atomic distances were also observed for Fe. At 700 °C, the Fe-Al in ω- Al_7_Cu_2_Fe had two groups in the first and second shells with N = 5 and N = 4 and distances of 2.441 Å and 2.473 Å, respectively. At 800 °C, these distances between Fe-Al changed to 2.589 Å and 2.713 Å, but the CNs did not change. After observing the liquid state at 800 °C, the heat was terminated, and the sample was cooled in the ultra-high vacuum to RT with a 50 °C/min cooling rate. At RT, the XRD graphs revealed new peaks—specifically, the QC phase. To better understand the process of QC formation during cooling, XAS data was collected at RT around the Cu and Fe K-edges. The results of the XAS measurements for R-space and k-space are shown in Fig. 5b–e. These data show radical changes to the structure of the local environment of Cu and Fe. Compared to the liquid state at 800 °C, the R-space at RT experienced a notable increase in the amplitude of |F(R)| (Fig. 5b,c), which could be explained as a crystallisation from liquid. The XAS data showed that from 800 °C to RT, the distance of the Cu-Al bond and Cu-Cu bond decrease around Cu atoms, which means that these two bonds have a stronger interaction at RT compared to the sample at liquid state. It also shows that the environment atoms around Cu at RT follow the same sequins shells as the liquid state at 800 °C (Table 2).

A comparison of these data shows that, in the first shell, Cu has the nearest distance with Al (2.533 Å) in ω- Al_7_Cu_2_Fe at 700 °C. At 800 °C, the distance of Al-Cu decreased to 2.441 Å, which indicates a stronger bond between Cu and Al in the first shell. In the second shell, the Cu-Cu atomic distance with N = 2 increased from 2.545 Å (at 700 °C) to 2.648 Å (at 800 °C). Moreover, the Cu-Cu bonding was in the second shell for the ω- Al_7_Cu_2_Fe at 700 °C, but when the sample reached the liquid state, a significant change in atomic distance was observed, and the Cu-Cu bonding was relocated to the forth shell. This observation suggests a weaker interaction between Cu-Cu in the liquid state compared to the crystal phase. Besides the relocation of the Cu-Cu shell, the Cu-Al in the third shell for ω- Al_7_Cu_2_Fe at 700 °C had an atomic distance of 2.579 Å with the CN 4 (N = 4), which split into two shells (second and third) at 800 °C with the equal CNs (N = 2) and distances of 2.575 Å and 2.590 Å, respectively.

A similar change in the atomic distances were also observed for Fe. At 700 °C, the Fe-Al in ω- Al_7_Cu_2_Fe had two groups in the first and second shells with N = 5 and N = 4 and distances of 2.441 Å and 2.473 Å, respectively. At 800 °C, these distances between Fe-Al changed to 2.589 Å and 2.713 Å, but the CNs did not change. After observing the liquid state at 800 °C, the heat was terminated, and the sample was cooled in the ultra-high vacuum to RT with a 50 °C/min cooling rate. At RT, the XRD graphs revealed new peaks—specifically, the QC phase. To better understand the process of QC formation during cooling, XAS data was collected at RT around the Cu and Fe K-edges. The results of the XAS measurements for R-space and k-space are shown in Fig. 5b–e. These data show radical changes to the structure of the local environment of Cu and Fe. Compared to the liquid state at 800 °C, the R-space at RT experienced a notable increase in the amplitude of |F(R)| (Fig. 5b,c), which could be explained as a crystallisation from liquid. The XAS data showed that from 800 °C to RT, the distance of the Cu-Al bond and Cu-Cu bond decrease around Cu atoms, which means that these two bonds have a stronger interaction at RT compared to the sample at liquid state. It also shows that the environment atoms around Cu at RT follow the same sequins shells as the liquid state at 800 °C (Table 2).

The fluctuation in atomic distance for Fe is more significant from 800 °C to RT. The XAS data collected at RT around Fe showed that at some point during the cooling process, the first (N = 5, 2.589 Å) and second shells (N = 4, 2.713 Å) had combined into a single shell with N = 9 and the atomic distance in the first shell was 2.489 Å. Increasing in the CN in the first shell from N = 4 to N = 9 and the shorter bond length at RT is evidence that Fe-Al has a stronger bond. Based on a study of X-ray photoemission, Belin *et al*.^43^ demonstrated that the Cu(3d) states shift the densities of Al(3p) and Fe(3d) states toward higher and lower binding energies, respectively; this is favourable for the Fe 3d–Al 3p hybridisation, leading to a strong bonding of Fe-Al.

The increased amplitude of the maxima of |F(R)| and shifting of k-space at RT compared to that at 800 °C show the formation of QCs (Fig. 5b–e). We can clearly see that the changes in the atomic distances and CNs are significant at RT and cannot be explained by crystalline approximation. These significant changes are the result of QC formation. In fact, in line with our results, previous works have demonstrated through simulations of the experimental K-Fe EXAFS function that the first coordination shell with N = 9 around Fe is an icosahedron^23,44^.

Yamamoto *et al*.^45^ showed the structure refinements of icosahedral Al-Cu-Fe QC using 6-dimensional cluster models and synchrotron radiation data from the least-squares refinements method^46^. They proposed that the first shells of Mackay-like clusters are highly disordered. In an illustration of the second and third shells of Mackay-like clusters, they demonstrated that one atom in the second shell is the center of the other cluster, while there are five atoms simultaneously located as a part of the third shell in another cluster (shown in Fig. 2 of ref.^45^). Our *in-situ* XAS results confirm this model. We show the precise atomic distance of Cu-Al(Fe) and Fe-Al bonding in QC at RT (Table 2). Using the atomic distances, CN and the atomic environment of QC, we were able to schematically illustrate each part of the second shell of Mackay-like clusters, as shown in Supplementary Fig. 3. Supplementary Fig. 3a,d show the atomic environment with CN = 9 around the Fe atom and the atomic environment around the Cu atom, respectively. A similar drawing of simulated Mackay-like clusters was also presented in Yamamoto *et al*., which clearly demonstrates the strong agreement between our experimental observation and the model proposed by Yamamoto *et al*.^45^.

## Conclusion

This study provides a dynamic perspective of the phases that evolve during the heating and cooling of an Al-Cu-Fe ternary thin films through *in-situ* XRD and XAS techniques. XAS techniques were used to give a clear picture of the atomic distance, CN and atomic environments of Cu and Fe. To verify the experimental data, the energy spectra for Cu and Fe K-edges were calculated by FDMNES simulation. The simulation confirmed the XAS data for η-AlCu θ-Al_2_Cu, β-Al(CuFe) and ω- Al_7_Cu_2_Fe phases at different temperatures. Based on the observation of the local rearrangement of atoms upon the formation of an icosahedral structure and by using a combination of *in-situ* XRD and *in-situ* XAS, we have proposed a model for the phase transition and QC formation mechanism during heating and cooling. We have demonstrated the important role that Cu-Cu and Fe-Al distance plays in the formation of the QC.

XAS data showed that to achieve the QC phase, the system needs to be heated to a liquid state, after which Cu relocates in the system at a high temperature. The EXAFS-XANES data shows that up until the sample was heated to 700 °C the shift of the atomic distance for the Cu and Fe K-edges were small. However, between 700 °C and 800 °C we observed a dramatic increase in atomic distance between Cu-Cu, in which Cu in the second shell relocated to the fourth shell. After reaching the maximum temperature of 800 °C, the liquid was cooled down to RT. It was during this cooling process that the QC phase formed by changing the atomic distances, CNs and atomic environments of Cu and Fe. When the QC was formed, significant changes were observed around Fe, specifically two shells combined into a single shell with smaller atomic distances. This study provides insight into the formation of the QC phase from a liquid state and offers a better understanding of the atomic arrangement involved in forming a QC phase, which can be useful in fine-tuning the synthesis of QC materials for industrial applications.

## Supplementary information

In-Situ observation of local atomic structure of Al-Cu-Fe quasicrystal formation
