# Supplementary material for: In-Situ observation of local atomic structure of Al-Cu-Fe quasicrystal formation
Source: Sci Rep. 2019 Feb 4;9:1245. doi: 10.1038/s41598-018-37644-x (PMC6362143; doi:10.1038/s41598-018-37644-x)
Supplement: Supplementary file 1 — In-Situ observation of local atomic structure of Al-Cu-Fe quasicrystal formation [file 41598_2018_37644_MOESM1_ESM.docx]

**Supplementary Figures and Table:**

**In-Situ observation of local atomic structure of Al-Cu-Fe quasicrystal formation**

Hadi Parsamehr^1^, Ying-Jiu Lu^2^, Tzu-Ying Lin^3^, An-Pang Tsai^4^, Chih-Huang Lai^1*^

^1^ Department of Material Science and Engineering, National Tsing Hua University, Hsinchu, 30013, Taiwan.

"*Correspondence to [[chlai@mx.nthu.edu.tw](mailto:chlai@mx.nthu.edu.tw)]"

^2^ National Synchrotron Radiation Research Center, Hsinchu 30076, Taiwan.

^3^ Department of Electrical Engineering, Tokyo University of Science, Japan.

^4^ Institute of Multidisciplinary Research for Advanced Materials, Tohoku University, Sendai 980-8577, Japan and National Institute for Materials Science, 305-0047 Tsukuba, Japan.


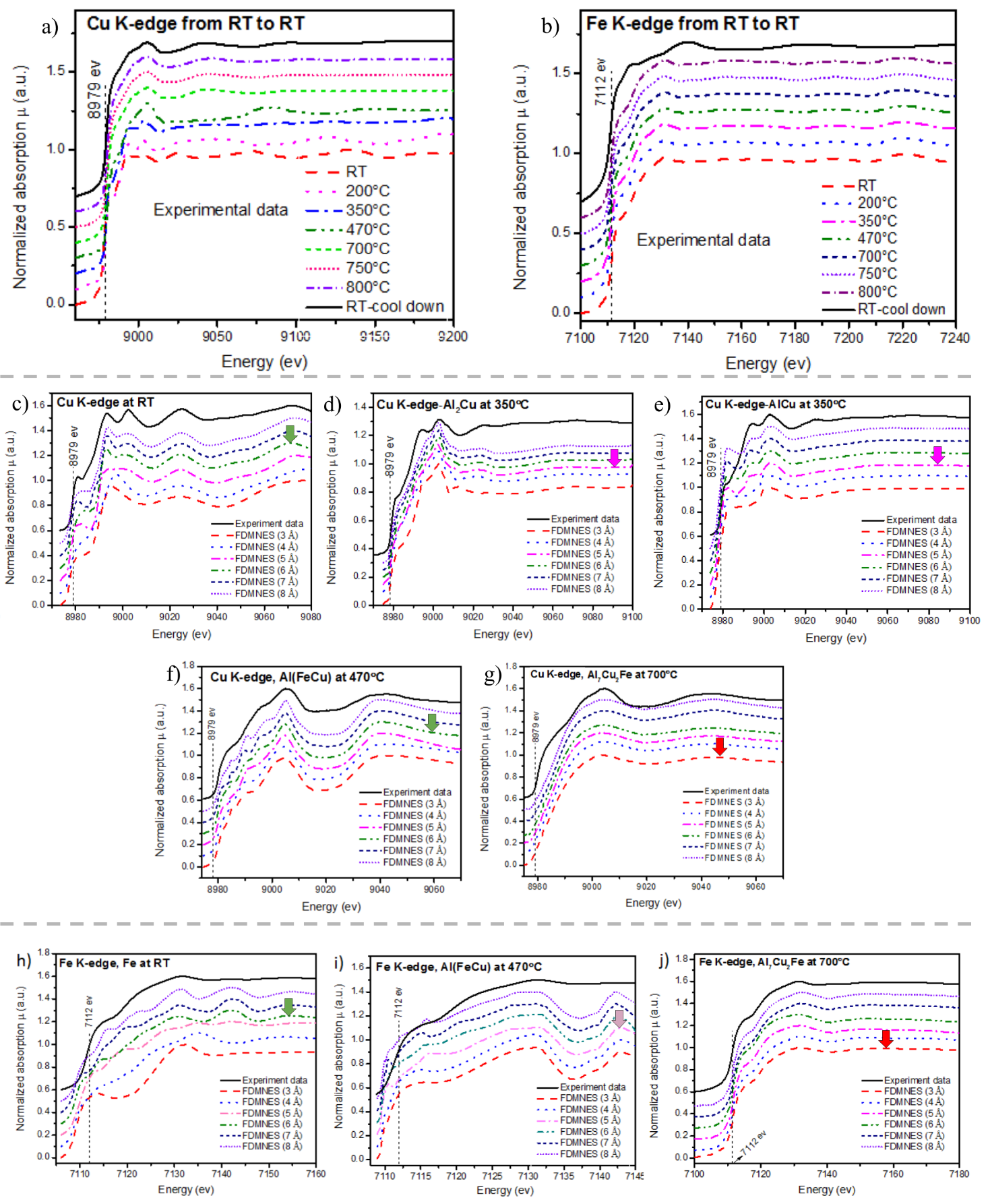


**Supplementary Figure 1**. Cu (a) and Fe (b) K-edges background subtracted and normalised XANES spectra of Al-Cu-Fe from RT (multilayer) to RT (quasicrystal) that initially temperature increased to 800°C. FDMNES results for Cu K-edge of copper at RT (c); for Cu K-edge of Al_2_Cu (d) and AlCu (e) phases at 350°C; for Cu K-edge of Al(CuFe) phase at 470°C (f); for Cu K-edge of Al_7_Cu_2_Fe phase at 700°C (g); for Fe K-edge of Fe at RT (h); for Fe K-edge of Al(CuFe) phase at 470°C (i); and for Fe K-edge of Al_7_Cu_2_Fe phase at 700°C (j).


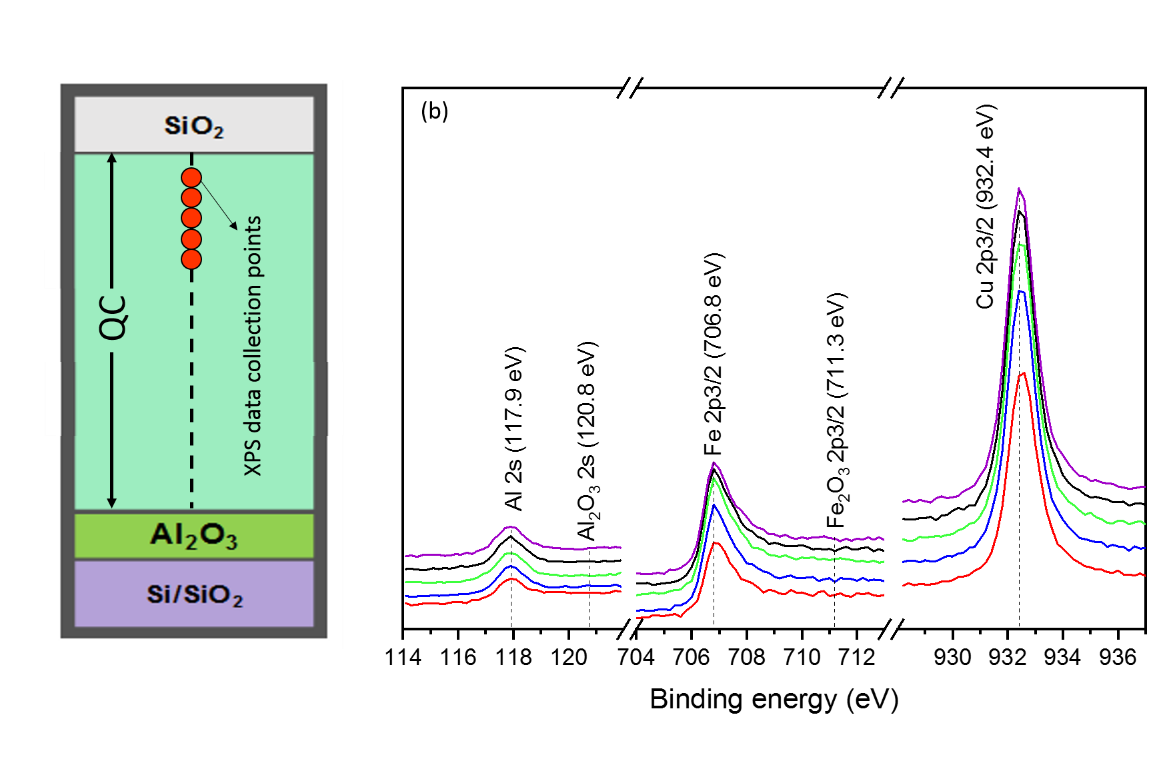


**Supplementary Figure 2.**  (a) The schematic diagram of the XPS collection points (five red circles); (b) XPS results for elemental Al, Cu and Fe after heat treatments.


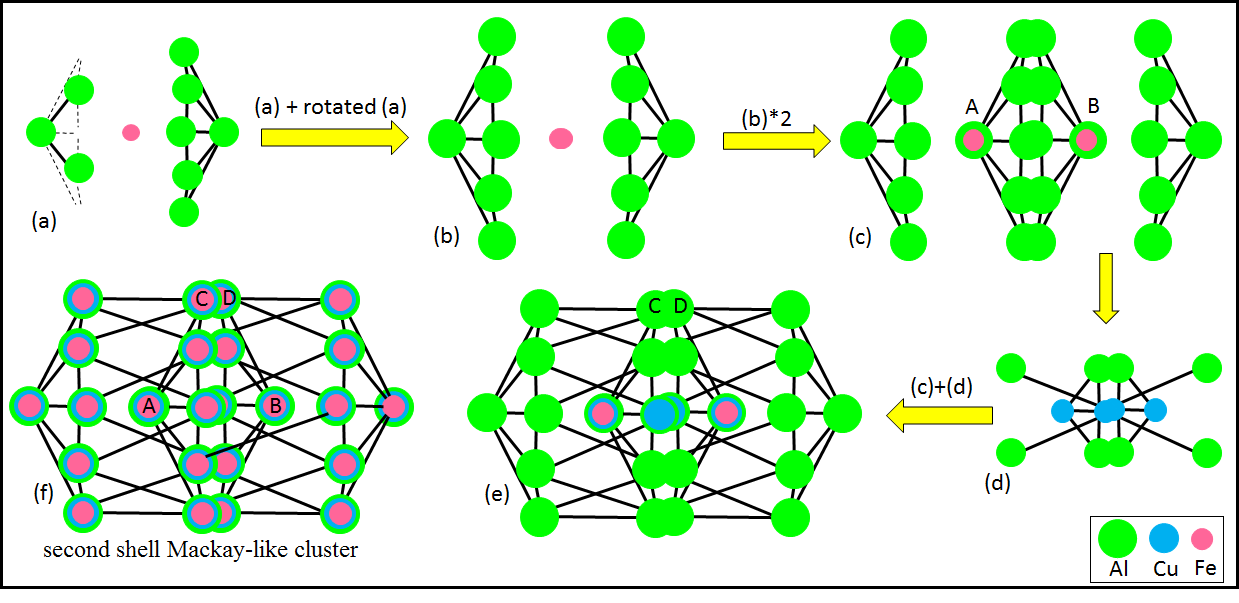


**Supplementary Figure 3.** The detailed schematic illustration of second shell Mackay-like cluster by using the QC data from in-situ XAS analysis. One atom in the second shell is the center of the other cluster [A and B in (c) and (f)]. Note that the positions of C and D atoms are the same as in (e) and (f).

**Supplementary Table 1**. Average bond distances for FCC Cu, BCC Fe, η-AlCu and θ-Al_2_Cu phases and CNs for the principal shells of atomic neighbours surrounding Cu and Fe centres. (R: atomic distance from Ref. [35], R*: atomic distance to neighbouring atom from the experimental data, N: CN, σ =R-R*, σ^2^: mean square relative displacement).

| K-edge | type of atom | N | R(Å) | R*(Å) | σ(Å) | σ^2^(Å) | K-edge | type of atom | N | R(Å) | R*(Å) | σ(Å) | σ^2^(Å) |
| --- | --- | --- | --- | --- | --- | --- | --- | --- | --- | --- | --- | --- | --- |
| FCC Cu- RT | Cu-Cu | 12 | 2.555 | 2.545  ±003 | 0.01 | 0.008 | BCC Fe at 200°C | Fe-Fe | 8 | 2.482 | 2.84  ±0.005 | 0.003 | 0.006 |
|  | Cu-Cu | 6 | 3.613 | 3.571  ±0.01 | 0.042 | 0.012 |  | Fe-Fe | 6 | 2.866 | 2.860  ±0.009 | 0.004 | 0.01 |
|  | Cu-Cu | 24 | 4.425 | 4.462  ±0.022 | 0.037 | 0.011 |  | Fe-Fe | 12 | 4.054 | 4.111  ±0.011 | 0.058 | 0.008 |
| BCC Fe-RT | Fe-Fe | 8 | 2.482 | 2.451  ±0.005 | 0.032 | 0.004 | θ-Al_2_Cu 350°C | Cu-Cu | 2 | 2.438 | 2.532  ±0.003 | 0.094 | 0.004 |
|  | Fe-Fe | 6 | 2.867 | 2.832  ±0.007 | 0.035 | 0.004 |  | Cu-Al | 8 | 2.590 | 2.714  ±0.031 | 0.124 | 0.07 |
|  | Fe-Fe | 12 | 4.054 | 4.041  ±0.01 | 0.013 | 0.009 | η-AlCu350°C | Cu-Cu | 2 | 2.534 | 2.504  ±0.004 | 0.030 | 0.005 |
| Cu at 200°C | Cu-Cu | 12 | 2.555 | 2.542  ±0.009 | 0.013 | 0.01 |  | Cu-Cu | 2 | 2.567 | 2.63  ±0.013 | 0.062 | 0.011 |
|  | Cu-Cu | 6 | 3.613 | 3.590  ±0.011 | 0.027 | 0.01 |  | Cu-Al | 2 | 2.59 | 2.578  ±0.017 | 0.012 | 0.018 |
|  | Cu-Cu | 24 | 4.425 | 4.420  ±0.015 | 0.05 | 0.012 | BCC Fe 350°C | Fe-Fe | 8 | 2.483 | 2.485  ±0.006 | 0.003 | 0.009 |
|  |  |  |  |  |  |  |  | Fe-Fe | 6 | 2.867 | 2.855  ±0.014 | 0.011 | 0.012 |
